# Renaissance for magnetotactic bacteria in astrobiology

**DOI:** 10.1038/s41396-023-01495-w

**Published:** 2023-08-17

**Authors:** Jianxun Shen, Greig A. Paterson, Yinzhao Wang, Joseph L. Kirschvink, Yongxin Pan, Wei Lin

**Affiliations:** 1 Key Laboratory of Earth and Planetary Physics, Institute of Geology and Geophysics, Chinese Academy of Sciences, Beijing, 100029 China; 2 France-China Joint Laboratory for Evolution and Development of Magnetotactic Multicellular Organisms, Chinese Academy of Sciences, 100029 Beijing, China; 3 Department of Earth, Ocean and Ecological Sciences, University of Liverpool, Liverpool, L69 7ZE UK; 4 State Key Laboratory of Microbial Metabolism, School of Life Sciences and Biotechnology, Shanghai Jiao Tong University, Shanghai, 200240 China; 5 Division of Geological & Planetary Sciences, Calfiornia Institute of Technology, Pasadena, CA 91125 USA; 6 Marine Core Research Institute, Kochi University, Kochi, 780-8520 Japan; 7 College of Earth and Planetary Sciences, University of Chinese Academy of Sciences, Beijing, 100049 China

**Keywords:** Biogeochemistry, Water microbiology

## Abstract

Capable of forming magnetofossils similar to some magnetite nanocrystals observed in the Martian meteorite ALH84001, magnetotactic bacteria (MTB) once occupied a special position in the field of astrobiology during the 1990s and 2000s. This flourish of interest in putative Martian magnetofossils faded from all but the experts studying magnetosome formation, based on claims that abiotic processes could produce magnetosome-like magnetite crystals. Recently, the rapid growth in our knowledge of the extreme environments in which MTB thrive and their phylogenic heritage, leads us to advocate for a renaissance of MTB in astrobiology. In recent decades, magnetotactic members have been discovered alive in natural extreme environments with wide ranges of salinity (up to 90 g L^−1^), pH (1–10), and temperature (0–70 °C). Additionally, some MTB populations are found to be able to survive irradiated, desiccated, metal-rich, hypomagnetic, or microgravity conditions, and are capable of utilizing simple inorganic compounds such as sulfate and nitrate. Moreover, MTB likely emerged quite early in Earth’s history, coinciding with a period when the Martian surface was covered with liquid water as well as a strong magnetic field. MTB are commonly discovered in suboxic or oxic-anoxic interfaces in aquatic environments or sediments similar to ancient crater lakes on Mars, such as Gale crater and Jezero crater. Taken together, MTB can be exemplary model microorganisms in astrobiology research, and putative ancient Martian life, if it ever occurred, could plausibly have included magnetotactic microorganisms. Furthermore, we summarize multiple typical biosignatures that can be applied for the detection of ancient MTB on Earth and extraterrestrial MTB-like life. We suggest transporting MTB to space stations and simulation chambers to further investigate their tolerance potential and distinctive biosignatures to aid in understanding the evolutionary history of MTB and the potential of magnetofossils as an extraterrestrial biomarker.

## Introduction

In 1996 McKay et al. [1] presented potential relic biogenic activity preserved in the Martian meteorite Allan Hills 84001 (ALH84001), discovered in the Allan Hills region of Antarctica in 1984. The meteorite, an ancient coarse-grained, cataclastic orthopyroxenite, contains a myriad of microscopic “disk-like” carbonate assemblages embedded within the fracture walls of the numerous cracks and fissures that pervade the ground mass. Intimately associated within and throughout these carbonate assemblages located within ALH84001, a population of nanocrystalline magnetites were identified [1, 2] that exhibited chemical and physical properties that bore a similarity to terrestrial intracellular magnetites produced by biomineralizing microbes, particularly the magnetotactic bacteria (MTB). These magnetites have a restricted size range with an unusual crystal morphology exhibiting an intact crystal lattice structure absent of heteroatom substitution and, in some instances, loosely organized into chain configurations. Taken collectively, in combination with accessory findings such as co-localization with simple organic species, these results were interpreted as potential biomarkers. Rock magnetic tests on the ALH84001 carbonate blebs limited the fraction of the magnetite crystals that might be aligned in chains to less than about 10% [3]. These purported similarities between terrestrial biogenic magnetite and the Martian magnetites in ALH84001 stimulated numerous debates within the scientific community, with some research teams suggesting that the nanoscale magnetites were the products of the partial thermal decomposition of the host carbonate due to impact or volcanic induced heating (e.g., [4]). This interpretation is inspiring but still remains contested [1, 2, 5].

While the origin of the ALH84001 magnetite crystals deserves further investigation it spurred the establishment of NASA’s Astrobiology program and generated significant interest in biomineralizing microbes, particularly MTB, as potential detection targets of extraterrestrial or early Earth’s life [6], and encouraged astrobiologists have widened the search for other biosignatures, such as organic matter, chemical biomarkers, and isotopic biosignatures. Although contemporary Mars lacks a magnetic field and thick atmosphere, early Mars was thought to be covered with liquid water and had a strong magnetic field that could protect the early atmosphere [7]. The Martian surface, as evidenced by Martian meteorites and remote sensing observations, was enriched in iron oxides, sulfates, and silicates. Based on our knowledge of extant terrestrial life, it is therefore critical to understand what types of organisms might plausibly have inhabited, and co-evolved, in early Mars-like environments. Inspired by recent findings that MTB can adapt to different facets of Mars-like extreme environments [8–10], we suggest revisiting astrobiology research on MTB in the recognition of their biological survivability in Mars analog environments.

In this Perspective, we discuss future prospects for this highly interdisciplinary and important field, and promising applications of MTB in astrobiology. First, the primeval emergence of MTB on Earth and the potential habitable conditions of early Mars are discussed. Second, we review the tolerance and responses of MTB members to natural and artificially simulated extreme environments that are similar to different geological periods on Mars. Third, we outline several practical signatures that could be used to detect ancient MTB on early Earth and potential MTB-like life on extraterrestrial bodies.

## MTB and Mars

**Fig. 1:**
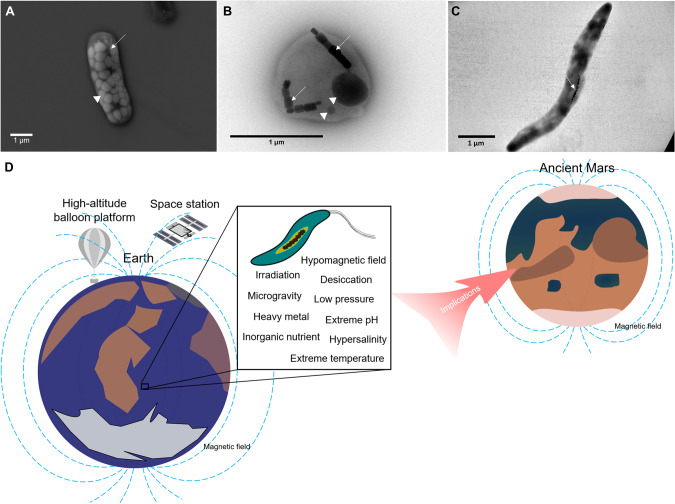
Illustrations of magnetotactic bacteria (MTB) under electron microscopes, and MTB from extreme environments on Earth and putatively ancient Mars. **A** The scanning electron micrograph and (**B**, **C**) transmission electron micrographs of MTB. White arrows indicate magnetosome chains, and white triangles indicate granules. **D** Terrestrial extremotolerant MTB and implications for ancient Mars. MTB are found to be able to resist various hostile settings, such as irradiation, hypomagnetic exposure, microgravity, metal stress, hypersalinity, acidic/hyperalkaline pH, and freezing/moderately hyperthermal temperatures. MTB emerged early in Earth’s history. Since ancient Mars was also characterized by many MTB-favorable aquatic/sediment oxic-anoxic conditions and a global magnetic field, it is proposed that Mars may have harbored MTB-like life in the past.

Armed with flagellar motility [11] or, occasionally, passive magnetotaxis [12], MTB synthesize intracellular magnetosome crystals that enable the cells to migrate along magnetic field lines (Fig. 1A–C). Magnetosomes are membrane bound crystals, typically composed of magnetite (Fe_3_O_4_), but sometimes greigite (Fe_3_S_4_) crystals. The monophyletic origin of MTB [13, 14], along with evidence against extensive inter-phylum and inter-class horizontal gene transfer of the gene cluster (the “magnetosome gene cluster, MGC”) that controls magnetosome formation, suggest that they are among the most ancient prokaryotes on Earth [13–15]. On the basis of phylogenetic analysis and molecular clock dating, MTB are thought to have evolved early on Earth in the mid-Archean Eon, possibly even earlier [15–17]. Their intracellular iron-mineral particles leave an excellent trace in the geological record, and are called magnetofossils [18]. In addition to being globally widespread and phylogenetically diverse, MTB possess a variety of phenotypes that allow them to survive in a wide range of planetary analog environments. On Earth, the currently known oldest microfossils are also associated with carbonate and magnetite-hematite globules in ferruginous sedimentary rocks [19], with paleoenvironments that probably met the requirements for biological magnetoreception [20].

Based on one of the oldest Martian meteorites, ALH84001 (~4.09 Ga), natural remanent magnetization indicates that Mars at about 3.9–4.1 Ga had formed a geodynamo that could generate a global magnetic field (~50 μT) similar to the present-day Earth (25–65 μT) [21], but gradually lost its magnetic shield [22, 23]. With a global magnetic field, the Martian atmosphere was protected from solar winds and enabled a thicker, warmer atmosphere containing CO_2_ and water vapor. Temperatures at the Martian surface during this time must have been sufficient to support liquid water, as there is ample evidence from Gale and Jezero craters, both of which are inferred to be ancient Martian lakebeds. In the former, Gale crater may have been the site of either a large lake that persisted for millions of years [24] or a handful of smaller lakes present for up to a few tens of thousands of years [25–27]. While at Jezero, features at the edges of the crater have been interpreted as river delta deposits that formed in an ancient lake basin during the Late Noachian or Early Hesperian epochs (~3.6–3.8 billion years ago). Suboxic interfaces within lake sediments composed of gradients of reduced iron and sulfide with oxygen would constitute a suitable environment for contemporary MTB; therefore, anaerobic or microaerobic MTB-like species, if they emerged, could have survived in redox-stratified crater lakes on early Mars [28–30].

As the first type of magnetosensitive and biomineralizing organisms, MTB are proposed to co-evolve with the hostile radiative subaerial environments on the Archean Earth possibly through exaptation, which shifted the function of magnetosome formation from either iron storage [31] or detoxification to magnetotaxis [32]. With magnetosomes, MTB were able to not only scavenge their intracellular free radicals but also guide themselves away from shallow to deeper subaqueous oxic-anoxic interface zones where neither radiation doses nor destructive oxygen species were plentiful [32, 33]. Due to the similarity of early Mars to early Earth, the emergence of MTB-like life on Mars is an intriguing possibility and of course needs more investigation (Fig. 1D).

## Stress tolerance of MTB

A variety of magnetotactic members have been identified from numerous natural environments, including aquatic environments, sediments, and waterlogged soils [10]. Amongst them, some cultivable species have been bred as type strains and used for stress exposure experiments. Different strains are sensitive to different stress factors (Table 1), nutrients, and dissolved oxygen levels [34, 35].

**Table 1:** Stress tolerance and response metabolisms of representative MTB members.

Ecological niche or experimental approach	Strain	Stress tolerance	Relevant mechanisms	References
Brackish desert spring in Death Valley, USA; Mono Lake, Soda Spring, and Armagosa Valley pond in California, USA	*Desulfamplus magnetovallimortis* strain BW-1, *Deltaproteobacteria* strains ML-1, ZZ-1, and AV-1	Hypersaline environment (10–90 g L^−1^)	Chemoorganoheterotrophic, sodium chloride-dependent, sulfate reduction	[36, 38, 39]
Comprida Lagoon in Brazil, Richmond Mine in California, acidic peatland in China	*Herbaspirillum* sp. CLV-1, MTB from *Nitrospirota*, *Proteobacteria*, *Desulfobacterota*, *Omnitrophota*, *SAR324*, *Fibrobacterota*, and *Planctomycetota*	Acidic environments (pH 0.8–5.7)	Intracellular acidic granules	[17, 42, 43]
Mono Lake, Soda Spring, and Armagosa Valley pond in California, USA	*Deltaproteobacteria* strains ML-1, ZZ-1, and AV-1	Hyperalkaline environment (pH 9.0–10.0)	Cytoplasmic buffering effect	[39, 44]
Admiralty Bay at King George Island, Antarctica	Magnetotactic cocci, vibrio, and bacilli	Low temperature (<1 °C)	/	[48]
Great Boiling Springs, Little Hot Creek, and Mickey Hot Springs, USA; Lake Miyun, China; Tengchong hot springs, China	*Candidatus Thermomagnetovibrio paiutensis* strain HSMV-1, *Nitrospirae* strain MHS-1, Magnetotactic cocci, *Nitrospirae* MTB	Moderately high temperature, temperature variation (9–70 °C)	Temperature tolerance through MTB community rearrangement	[51–54, 94]
Mickey Hot Springs, USA	*Nitrospirae* strain MHS-1	Arsenic stress (~1 mg L^−1^)	Magnetosome arsenic scavenging	[53]
Near space exposure	*Magnetospirillum gryphiswaldense* strain MSR-1	Desiccation, irradiation, low temperature	Magnetosomes raise survival rates	[9]
UV-B irradiation	*Magnetospirillum magneticum* strain AMB-1, *Magnetospirillum* sp. XM-1	Irradiation (2.0 10.8 W m^−2^) genetic damage and intracellular reactive oxygen species	Oxidative stress and DNA damage repair systems	[59, 60]
Visible light irradiation	*Magnetospirillum magneticum* strain AMB-1	Irradiation (45 μmol photons m^−2^ s^−1^)-triggered intracellular reactive oxygen species	Upregulated magnetosome formation, downregulated antioxidant enzyme and DNA repair genes, photoreceptors	[63]
Magnetic shielding device	*Magnetospirillum magneticum* strain AMB-1, *Candidatus Magnetococcus massalia* strain MO-1	Hypomagnetic field (2–500 nT)	Unaffected or upregulated magnetosome synthesis, enhanced biomineralization	[66, 67]
Space Shuttle and Space Station exposure	*Magnetospirillum magnetotacticum* strain MS-1	Microgravity (~0 m s^−2^)	Gradual loss of magnetotaxis	[68]
Metal-enriched culture media	*Magnetospirillum gryphiswaldense* strain MSR-1, *Magnetospirillum magneticum* strain AMB-1, *Desulfovibrio magneticus* RS-1	Heavy metal stress (Co, Ni, Mn, Zn, Cu, Se, Cd, and Te)	Metal storage organelle	[70, 73–75]

## Natural extreme environments

Various natural ecosystems are characterized by adverse conditions such as extreme salt concentrations, extreme pH, and extreme temperatures that are analogous to planetary environments. Studies in these environments identified new populations of MTB and enlarged the MTB phylogenetic taxonomy and natural habitats. It is reasonable to expect that the taxonomic diversity and ecological niche of MTB can be further expanded our understanding of these extreme environments [8, 10].

### High salinity tolerance

MTB are able to inhabit water columns or chemically-stratified sediments with a broad range of salinity [36, 37]. From the brackish (26 g L^−1^ salinity) Badwater Basin spring in the extremely arid Death Valley, USA, the *Desulfamplus magnetovallimortis* strain BW-1 was isolated [38]. A large rod-shaped MTB species was discovered from Salt Pond (30 g L^−1^ salinity) in Woods Hole and a red-colored pool (52 g L^−1^ salinity) near Salton Sea, USA [36]. BW-1 is a strictly anaerobic chemoorganoheterotrophic strain that is capable of reducing sulfate to form magnetite and/or greigite magnetosomes, which is a halophile that requires more than 10 g L^−1^ of sodium chloride to grow [38]. Strikingly, MTB from Mono Lake can even tolerate hypersalinity up to 75–90 g L^−1^ [39].

Briny subglacial lakes and thin shallow subsurface liquid layers have been detected on Mars [40, 41]. Due to the high contents of soluble salts, these waters are expected to be hypersaline. Whether MTB could adapt to this adverse condition needs further investigation. Certainly, water bodies with low salinity likely existed on early Mars [30].

### Extreme pH tolerance

Acidotolerant MTB with a variety of shapes were discovered in the acidic Comprida Lagoon (pH 4.4–4.9), Brazil [42], and waterlogged acidic peatland soils (pH 4.3–5.7), China [17]. Intracellular acidic granules were found in MTB as a mechanism to neutralize cytoplasm [42] (Table 1). Moreover, MTB observed by cryogenic transmission electron microscopy and transcriptomic analyses can tolerate the extremely low pH (0.8–1.8) conditions in acidic mine drainage at Iron Mountain, USA [43].

Previously, alkaliphilic MTB have been isolated from the hyperalkaline (pH 9.0–10.0) Mono Lake, Soda Spring, and Armagosa Valley in California, USA [39]. These alkaliphiles likely keep their cytoplasmic neutrality by storing aqueous pH buffers [44] (Table 1). They also evolved to be able to perform iron uptake when iron species are more easily precipitated under conditions typically viewed as unfavorable for MTB.

Martian soils are heterogeneous in terms of physicochemical characteristics due to the lack of aqueous dissolution. Hence, acidic aquatic environments and alkaline soils both existed on Mars globally and locally [45, 46]. On early Mars, ancient lakes such as the one at Gale crater were characterized by circumneutral pH, which would have been suitable for a broad range of species if they ever existed on Mars [26, 47].

### Extreme temperature tolerance

In the severe Antarctic circumpolar environments, active magnetotactic cocci within the *Proteobacteria* were observed in different years from multiple oceanic sediments around Admiralty Bay, King George Island, indicating the existence of psychrophilic MTB members [48]. In polar regions, the high strength and steep inclination of the local geomagnetic field provide additional recompenses to MTB. According to paleoenvironmental records in the early Cenozoic, these MTB can utilize more bioavailable eolian-derived iron-bearing dust from Antarctica [49].

Although the present-day Martian surface is extremely cold, many ancient Martian hydrothermal vents or hot springs have been recognized [50]. On Earth, hot springs (30–70 °C) are found to be inhabited by thermophilic MTB species, most commonly *Nitrospirae* [51–54]. On this basis, MTB could have evolved to tolerate or thrive within geothermal fields that may have occurred on ancient Mars and deep-subsurface moderate hydrothermal vent-like structures, which may exist on Mars over geologic time [55].

### Earth’s near space exposure

With multiple natural extreme conditions (extreme dryness, ionizing radiation, low temperature, and low atmospheric pressure), the lower near space of Earth serves as an excellent Mars-like setting to study the stress tolerance of MTB [56, 57]. Intriguingly, after hours of exposure to the lower near space environment at 23 km, a considerable fraction (>12%) of the wild-type *Magnetospirillum gryphiswaldense* strain MSR-1 (MSR-1; isolated from freshwater sediments in the Ryck River, Germany) survived near space exposure [9]. This indicates that MTB may be able to survive interplanetary travel that may take terrestrial life to other astronomic bodies or bring life from extraterrestrial sources to the prebiotic Earth [58].

## Artificially simulated extreme environments

In addition to natural extreme environments, laboratory-based artificially simulated conditions can provide extra valuable insights into the impacts of controllable single environmental factors (e.g., irradiation, weak magnetic field, low gravity, and heavy metal enrichment) on MTB cultures.

### Radiation tolerance

Radiation is generally the most lethal threat to life in space. Wang et al. found that *Magnetospirillum magneticum* strain AMB-1 (AMB-1; a type-strain isolated from a freshwater sediment in Tokyo, Japan) produced larger magnetite crystals and longer magnetosome chains after UV-B (275–330 nm) exposure (2.0–10.8 W m^-2^), while genetic lesions and free radicals accumulated within cells and cell growth was inhibited with increased radiation dose [59]. After a period of recovery, the intracellular levels of lesions and free radicals decreased back to normal, implying the presence of radiation-induced damage repair mechanisms [59, 60] (Table 1). Magnetosomes can act as peroxidase-like catalysts and help to eliminate reactive oxygen species [33, 61], and a study using *Magnetospirillum* sp. XM-1 (a strain isolated from a city moat in Xi’an, China) showed interactive effects between UV radiation and oxygen stress [62]. Under visible light illumination (cold light source with an intensity of 45 μmol photons m^−2^ s^−1^), magnetosome formation and photoresponse activities of AMB-1 were upregulated, accompanied by a decline of intracellular reactive oxygen species [63] (Table 1). Additionally, the peroxidase-like activity of magnetosomes was enhanced by visible light irradiation [64].

### Hypomagnetic field

On present-day Mars, the regional magnetic fields from crustal magnetic anomalies are generally a few to hundreds of nano-Tesla (nT) [23], while the InSight lander determined the crustal field of its landing site can be as large as 2 µT [65]. Previous studies found that hypomagnetic field (500 nT for 16 h) triggered AMB-1 to upregulate the magnetosome biomineralization, while maintaining the expression of iron transport genes [66]. The growth of a marine ovoid-coccoid MTB *Magnetococcus massalia* strain MO-1 (isolated from sediments of the Mediterranean Sea) was stunted by hypomagnetic field of 2 nT within 4 days, while the regular formation of magnetosomes was not affected [67] (Table 1).

### Low gravity environments

The gravity of all Earth-like rocky planets and moons in the Solar System is less than that of Earth (9.807 m/s²). For example, the surface gravity of Mars is 3.721 m/s². Two previous investigations of the effect of low gravity on *M. magnetotacticum* strain MS-1 (isolated from the freshwater Cedar Swamp in Woods Hole, USA) were done by loading samples in a magnetic field-controlled apparatus onboard the Space Shuttle Endeavour at 278–287 km altitudes for 4–5 days, and on the Space Shuttle Atlantis-Space Station Mir at 368–392 km altitudes for 120 days [68]. Results suggested that a microgravity environment could impair microbial magnetotactic response (Table 1), and magnetosomes might additionally act as gravity sensors. Possibly in early Mars-like aquatic environments with necessary magnetosome-forming elements, MTB-like life may evolve to produce magnetosomes of differing sizes, shapes, and arrangements to adapt to ancient Martian environments.

### Heavy metal stress

Martian soils are found to be enriched in some heavy metals, including manganese (Mn), cobalt (Co), nickel (Ni), copper (Cu), zinc (Zn), gallium (Ga), and germanium (Ge) [69]. A previous medium-enrichment study found that magnetosomes from MTB, such as MSR-1, were capable of incorporating Co, Mn, Zn, and Cu into their interior and facilitating transportations of Ni, Mn, Zn, and Cu into other intracellular compartments (Table 1), resulting in an improvement of MTB tolerance to heavy metal-rich environments [70]. Incorporation of Co can even enlarge the size and length of magnetosomes and their magnetic coercivity, offering better magnetotaxis [71, 72]. MTB are additionally capable of recovering selenium (Se), cadmium (Cd), and Tellurium (Te), making them more tolerant of heavy metal-rich environments [73–75].

## Detection of MTB-like biosignatures

**Fig. 2:**
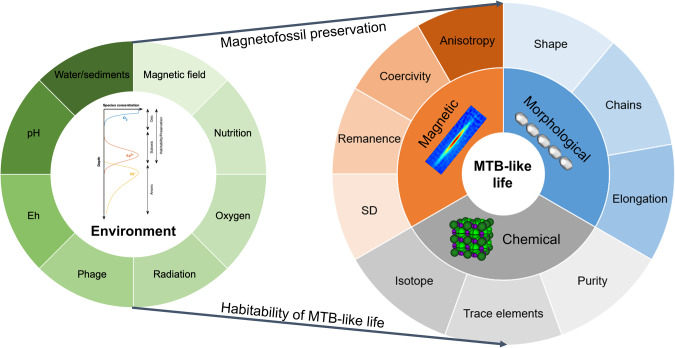
Schematic diagram of key factors in the search for MTB-like biosignatures. The environmental context ought to be suitable for the growth of MTB-like life, including an appropriate magnetic field >~ 6 µT [66, 67, 95], nutrient enrichment, oxygen content, pH range, redox stratification, irradiation condition, and potential phage interaction. Some common biosignatures of MTB-like life include magnetosome morphology, magnetic properties, and chemistry. Morphological biosignatures include distinct crystal morphology (e.g., elongated hexagonal prismatic magnetite with faceted ends), particle elongation, and particle chains. Magnetic biosignatures include single-domain (SD) sized particles, remanence, coercivity, and anisotropy. Chemical biosignatures include intact crystal lattice that is relatively free of defects (though it may occasionally have [111] twinned crystals), chemically pure composition from selective transport of iron, trace elements that are selectively incorporated into magnetosomes, and isotopes of relevant elements in magnetosomes. Note that a good biosignature is something difficult to produce through inorganic processes; the MTB-produced magnetosome chain structures display the effect of *Natural Selection* for magnetotaxis, with each of the magnetic, morphological, and chemical features being driven by selection to maximize the efficiency of the cellular magnetotactic response. The central intersection of these factors is what makes magnetofossils a superb biomarker [6].

The formation of chain arrangements of iron oxides or iron sulfides allows MTB to leave the fossil remains of magnetic particles, which can be preserved in the geologic record for billions of years [18, 76]. The ordered spatial organization of nanosized magnetite particles is somehow indicative of the preservation of a thermodynamically unfavorable state that most often requires biological production. (Random assemblages of magnetite crystals fall into clumps, not chains [77].) In addition to morphological microstructure or microtexture biosignatures, more technologies can be employed to support any future finding of magnetosome-like nanostructures in paleoenvironments favorable for MTB-like life and preservation of magnetofossils (Fig. 2).

### Morphological biosignatures

The size and arrangement of magnetite crystals within MTB are special and can be used as a biomarker to distinguish them from abiogenic particles. As crystal lattice defects interrupt the alignment of the Bohr Magnetons, decreasing the net magnetization, natural selection should lead to defect-free crystals. Similarly, the [111] crystallographic axis is the easy axis of magnetization, and elongation along this direction yields a more stable magnetization. If an assemblage of magnetite nanoparticles are characterized by this crystallographic perfection, elongate cuboctahedral configuration, and are associated intimately with organic compounds [76], then they are likely derived from MTB-like life. Further research on MTB crystalline structures and their preservation capacities are required. Based on natural selection in terms of maximizing cellular magnetic moments, MTB should have evolved to consume iron sources and form crystals in an efficient manner. These selected phenotypes of MTB-synthesized magnetosomes should permit MTB to align themselves along the magnetic field and avoid exposure to harsh subaerial conditions more biomechanically efficiently by adjusting their shapes [76].

### Magnetic biosignatures

MTB-produced magnetite crystals tend to have restricted width-length ratios and volumes that place the crystals in the single-domain (SD) magnetic size range; to take advantage of the physical effects provided by the geomagnetic field, MTB organize their magnetosomal chains that optimize the magnetic moments of the magnetite crystals [6]. Biological specific preferences of selection and arrangement of magnetic particle chains contribute to magnetic properties (e.g., coercivity, magnetic remanence, and magnetocrystalline anisotropy) that are different from abiotic sources [78]. First-order reversal curves (FORCs) and low-temperature data can additionally act as key tools for the characterization of organized chain structures composed of magnetic materials [10].

### Isotopic biosignatures

As biogeochemical iron cycling drivers, MTB leave isotopic fractionation traces (depletion in ^56^Fe/^54^Fe ratios, and mass-independent fractionation in ^57^Fe but not in ^54^Fe, ^56^Fe, ^58^Fe) in synthesized magnetite [79, 80]. In addition, oxygen isotopes in MTB magnetite crystals fractionate with ambient water molecules with a temperature-dependence [81]. By comparing iron and oxygen isotopes in the magnetite chains of interest with those in iron and moisture sources, potential traces of magnetite-forming MTB activity might be discovered if similar biologically driven isotope effects were detected. In addition, Mössbauer spectroscopy is a powerful tool for detecting iron-containing biomolecules. Iron isotope (^57^Fe)-based Mössbauer spectroscopy reveals possibly biologically characteristic features of the valence of iron elements, mineral types, and organics [82].

### Trace element and biogeochemical biosignatures

Magnetosomes incorporate trace elements in magnetite or greigite [70] that involve biological activities that imprint specific isotope ratios in these crystals. The diversity and significantly lower abundance (<1%) of incorporated elemental species within magnetite chains can be potential indicators of their biogenesis [83]. In addition, a coupled C-N geochemical signal can serve as a unique signature of biomineralized magnetite nanocrystals instead of the abiogenic or biomimetic counterpart [84]. Investigations of the chemical and isotopic compositions of trace elements in magnetosomes and abiogenic magnetite will shed light on the uniqueness of biologically produced iron particles.

### Phage infection biosignatures

Apart from MTB-driven iron cycling, bacteriophages that infect MTB have been proposed recently to be involved in the dynamics of the global iron cycle [85]. This is via transduction of biomineralization genes and regulation of host cell lysis, thereby increasing the release of magnetosomes (magnetofossils) into the environment during their life cycle [85]. These processes are not sufficiently studied and remain poorly understood. Potential interactions between MTB and phages are waiting to be discovered. Viruses are organic biochemical machines that are categorically located somewhere between life and prebiotic chemistry [86]. Thus, they could plausibly predate the last universal common ancestor of all living things (LUCA), either on Earth or in extraterrestrial habitable environments. Hence viruses may have co-existed with other organisms during the entire history of life on Earth [87]. MTB-associated phages and their interactions may influence biosignatures and affect characteristics such as morphological malformations, compositional chemical alterations, release rates of iron, accumulation of virion-like peptides, and associated changes in magnetic properties and spectral characteristics.

## Conclusions and prospects

Studies of magnetotactic extremophiles might help us understand potential life forms on early Earth and other astronomical bodies that have both strong magnetic fields and aquatic settings. To understand the potential applications of MTB in astrobiology, we suggest investigating MTB in early Earth-like and Mars-like extreme environments, and inspecting their genotypic and phenotypic functions. These studies might include their adaptation strategies and imprinted isotopic signatures on simple inorganic elements (e.g., Fe, S, N, Si, triple oxygen isotopes in magnetites, and quadruple sulfur isotopes in greigite magnetosomes). Furthermore, MTB strains could be transported to space environments on the Tiangong Space Station, International Space Station, or space environmental simulation chambers to study their survivability and strategies under Mars-like or other extraterrestrial conditions or impact-related shockwave scenarios [88, 89].

Despite the diversity of MTB detected from various extreme natural or artificial environments, specific genomic, transcriptomic, proteomic, and metabolomic analyses of relevant stress response pathways or mechanisms are rarely investigated. However, it is noteworthy that genomics alone may not provide unambiguous conclusions on the discovery of MTB [90]. Thus, we suggest using multiple omics techniques combined with electron microscopies and magnetic measurements to comprehensively understand the adaptation strategies of MTB and possibly MTB-virus interactions in extreme environments.

MTB were certainly involved in the iron cycling on Earth, both at present and in the past. However, the cycling paradigm has not been built. It is necessary to incorporate MTB into global models of iron cycling and investigate their isotopic fractionation in more detail (e.g., Fe fractionation in different strains and habitats; O and S isotopes in biogenic magnetite and greigite nanoparticles, respectively) to better understand potential isotopic biosignatures that may be utilized on Mars and to determine the earliest terrestrial MTB records from ancient rock materials [79].

As discussed above, early Martian MTB-like life is not entirely implausible, and if they were present, they could have readily acclimatized to the suboxic or oxic-anoxic transition zones in early Mars aquatic ecosystems (e.g., Gale crater lake and Jezero crater lake). Beyond Mars, the brackish oceans that may exist under the surface of icy moons, and numerous organic molecules within them [91], may also be a suitable environment for MTB. For example, Jupiter’s moon Ganymede is the largest moon in the Solar System, and one of the most favorable icy bodies to MTB-like life, due to the presence of an active dynamo and global magnetic field [92]. Ganymede has a very tenuous atmosphere, which is mainly composed of oxygen and a minor atmospheric constituent (nitrogen, hydrogen, and water vapor) [93]; thus, redox stratification likely occurs in subsurface liquid.

Considering the booming growth of international space explorations, several Mars (e.g., Perseverance, ExoMars, Tianwen-1, Tianwen-3, TEREX-1, and Mangalyaan 2) and Jovian system (e.g., Juno, JUICE, Europa Clipper, and Tianwen-4) missions are in action or preparation. To detect MTB-like biosignatures in situ or through sample return missions, we recommend using reflectance and Raman spectroscopies to identify magnetite or greigite minerals as well as organic compositions; using Mössbauer spectroscopy to speciate iron-bearing molecules; using trace element analyzers (e.g., X-ray fluorescence, atomic absorption spectroscopy, neutron activation analysis, proton-induced X-ray emission) to quantify trace elements within iron-bearing minerals; and using mass spectrometry to measure isotope ratios of elements and analyze structures of organic molecules. When sample return missions are successful, high-resolution electron microscopy can be readily applied to characterize configurations of magnetite or greigite crystals. More complicated liquid and gas chromatograms can be applied to identify and quantify organic species such as amino acids and nucleotides. Techniques used for biogeomagnetism can be applied to understand the magnetic properties of postulated MTB-like organisms or fossils. To prepare for these exciting, but practical investigations, we suggest that it is imminent to develop more advanced and reliable abovementioned scientific instruments.

## Data Availability

Data sharing not applicable to this article as no datasets were generated or analyzed during the current study.
